# Case Report: Coexistence of Anti-Glomerular Basement Membrane Disease, Membranous Nephropathy, and IgA Nephropathy in a Female PatientWith Preserved Renal Function

**DOI:** 10.3389/fphar.2022.876512

**Published:** 2022-06-01

**Authors:** Wei Qu, Nan Liu, Tianhua Xu, Binyao Tian, Meng Wang, Yanqiu Li, Jianfei Ma, Li Yao

**Affiliations:** Department of Nephrology, The First Hospital of China Medical University, Shenyang, China

**Keywords:** anti-glomerular basement membrane disease, membranous nephropathy, IgA nephropathy, immunotherapy, case report

## Abstract

The coexistence of anti-glomerular basement membrane (GBM) disease, idiopathic membranous nephropathy (IMN), and IgA nephropathy in one patient is a very rare case, which has not yet been reported. Whether the three diseases are correlated and the underlying mechanism remain unknown. Herein, we report a 48-year-old female patient that was admitted because of proteinuria and abnormal renal function, which was diagnosed as anti-GBM disease, idiopathic membranous nephropathy, and IgA nephropathy by renal biopsy. The patient received treatment including high-dose methylprednisolone pulse therapy, plasma exchange, and intravenous infusion of both cyclophosphamide (CTX) and rituximab. In the follow-up, the titer of the anti-GBM antibody gradually decreased, renal function was restored, and urinary protein was reduced, without significant adverse effects.

## Introduction

Anti-glomerular basement membrane (GBM) disease, an autoimmune glomerular disease, develops in genetically susceptible individuals exposed to some environmental factors (McAdoo and Pusey, 2017). It is characterized by linear deposition of immunoglobulin G (IgG) along the GBM. Anti-GBM disease is histologically associated with extensive crescent formation and clinically with rapidly progressive glomerulonephritis (RPGN) (Kojima et al., 2019). Membranous nephropathy (MN) is the most common cause of nephrotic syndrome in the elderly, characterized by subepithelial immune complex deposition along the GBM (Khorsan et al., 2019). IgA nephropathy is one of the most common autoimmune glomerular diseases worldwide, which features significant IgA1 deposition in the glomerular mesangium. It manifests as symptoms from asymptomatic mild hematuria and proteinuria to rapidly progressive crescentic glomerulonephritis (Khorsan et al., 2019).

Previous research has reported concurrent anti-GBM disease and MN, coexistence of anti-GBM disease and IgA nephropathy, and concurrent MN and IgA nephropathy. However, the coexistence of the three in one patient has not yet been reported. Herein, we report a patient with a coexisting anti-GBM disease, MN, and IgA nephropathy, which have been successfully treated.

## Case Presentations

A 48-year-old female patient developed symptoms such as low-grade fever, cough, and fatigue after catching a cold 1 month before admission. Half a month before admission, medical examination showed serum creatinine of 0.9 mg/dl, hemoglobin of 10.8 g/dl, serum albumin of 3.8 g/dl, and quantitative urinary protein excretion of 1.1 g/d; 1 week before admission, the serum creatinine level was 1.47 mg/dl, serum albumin was 3.4 g/dl, and the patient was positive for the anti-GBM antibody. She was admitted in April 2021, when her urine output was within the normal range, and she had no low-back pain, gross hematuria, eyelid edema, and edema of the lower extremities. The patient did not report fever, chest distress, shortness of breath, rash, and significant change in body mass and denied a medical history of sinusitis, hypertension, and diabetes mellitus. Laboratory examination indicated quantitative urinary protein excretion of 1.0 g/d, urinary protein 1+, urinary alpha-1-microglobulin (a1-MG) of 1.57 mg/L, microalbumin (MA) of 27.5 mg/dl, urine transferring (TRU) of 1.53 mg/dl, urine immunoglobulin-G (IgU) of 4.34 mg/dl, red blood cells of 222.62/HPF, and abnormal morphology reaching 80%. In addition, the total serum protein (6.23 g/dl), serum albumin (3.09 g/dl), serum creatinine (1.74 mg/dl), estimated glomerular filtration rate (eGFR) (47.7 ml/min), hemoglobin (9.7 g/dl), C-reactive protein (5.91 mg/dl), and anti-GBM antibody (551 U/ml, using the ELISA method) were also measured. The patient was negative for both the antineutrophil cytoplasmic antibody and the antibody to M-type phospholipase A2 receptor (PLA2R) (4.13 RU/ml, using the ELISA method) in serum; serum C3 and C4 levels were normal. No abnormality was found in the electrocardiogram, computerized tomography of the lung, and cardiac and abdominal ultrasonography.

**FIGURE 1:**
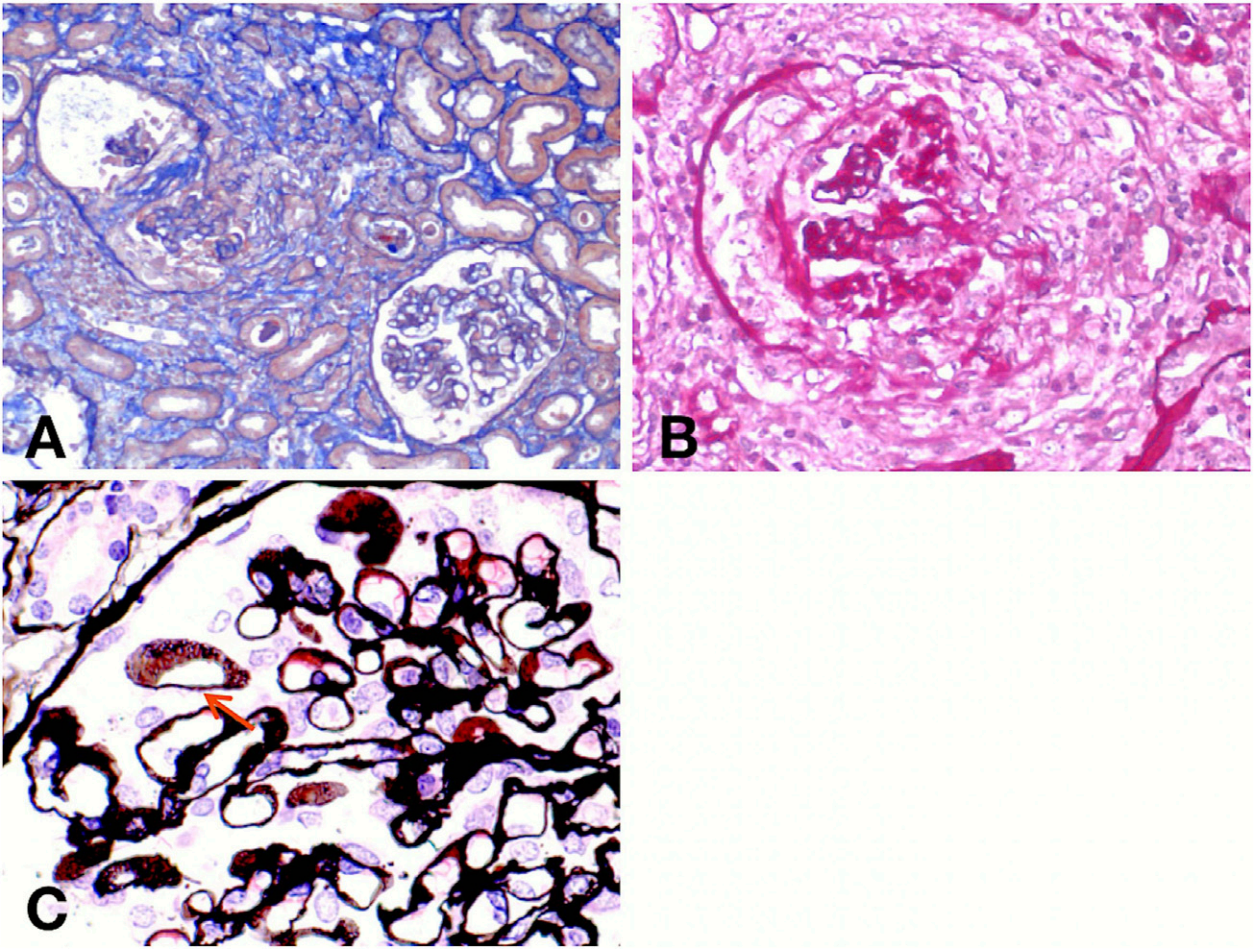
Light micrographs. Masson **(A)** and periodic acid–Schiff (PAS) **(B)** staining show crescent formation, fragmented capsule walls, and interstitial inflammatory infiltration. Periodic Schiff-methenamine silver (PASM) **(C)** staining reveals the formation of short subepithelial spikes (red arrow).

**FIGURE 2:**
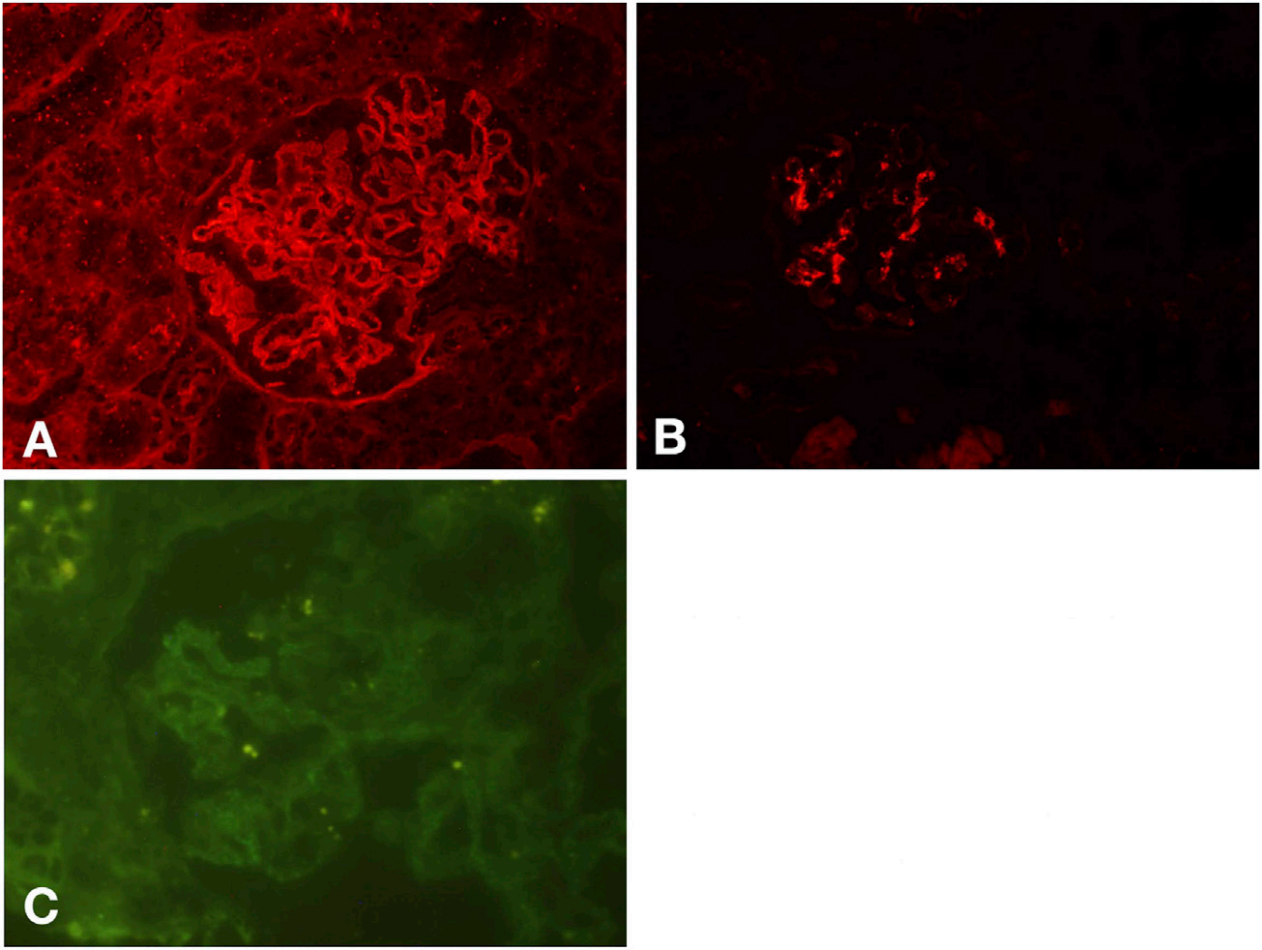
IF results. Fine granular deposition of IgG along glomerular capillary walls and segmental while weak line-like change areas of some glomerular capillary wall and glomerular capsule wall due to IgG **(A)**. Lumpy deposition of IgA in the mesangial area **(B)**. Fine granular deposition of PLA2R along glomerular capillary walls **(C)**.

**FIGURE 3:**
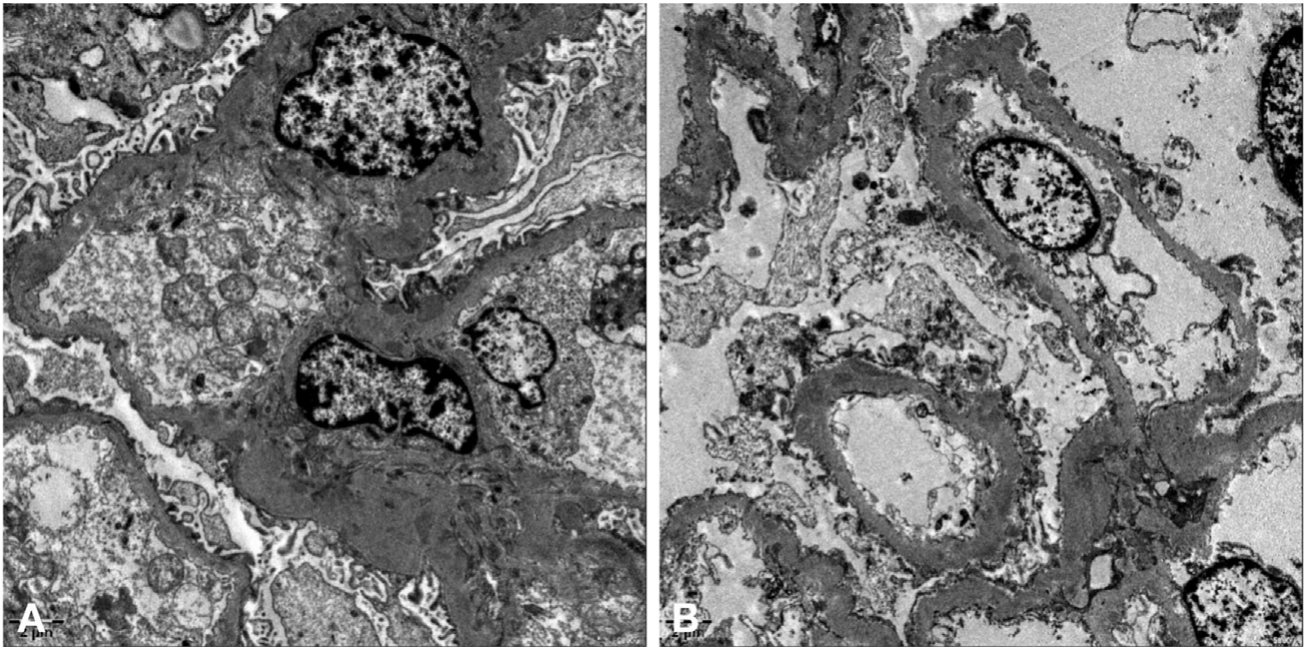
Electron micrographs. Mild, irregular thickening of the GBM and electron-dense deposits in the subepithelial side of the GBM **(A,B)**.

A renal biopsy was performed. Light microscopy showed global sclerosis (with evidence in the form of crescent formation revealed) in three, formation of large fibrous crescents in two, and segmental fragmentation of capsule walls accompanied by necrosis of capillary loops in two, of 16 glomeruli. Diffuse thickening of the GBM and mild proliferation of mesangial cells and matrix were also observed, which also revealed multi-focal (25%–50%) atrophy of renal tubules and multi-focal (25%–50%) mononuclear cell infiltration in the renal interstitium. Formation of short subepithelial spikes, podocyte swelling, and vacuolar degeneration were also observed (Figures 1A–C). Immunofluorescence (IF) demonstrated fine granular deposition of IgG (+++), PLA2R (+), THSD7A (−), IgG1 (++), IgG2 (−), IgG3 (−), IgG4 (+++), C1q (−), IgM (−), Fib (−), and C4 (−) along glomerular capillary walls; segmental while weak line-like change areas of some glomerular capillary wall and glomerular capsule wall due only to IgG; and lumpy deposition of IgA (+++) and C3 (++) in the mesangial area (Figures 2A–C). Mild, irregular thickening of the GBM (about 300–900 nm) was observed by electron microscopy, together with swelling of glomerular epithelial cells and vacuolar degeneration. Electron microscopy also showed diffuse foot process fusion, electron-dense deposits in the subepithelial side of the GBM, responses in the GBM, and mesangial cell and matrix proliferation. Electron-dense deposits were observed in the mesangial area (Figures 3A,B).

**FIGURE 4:**
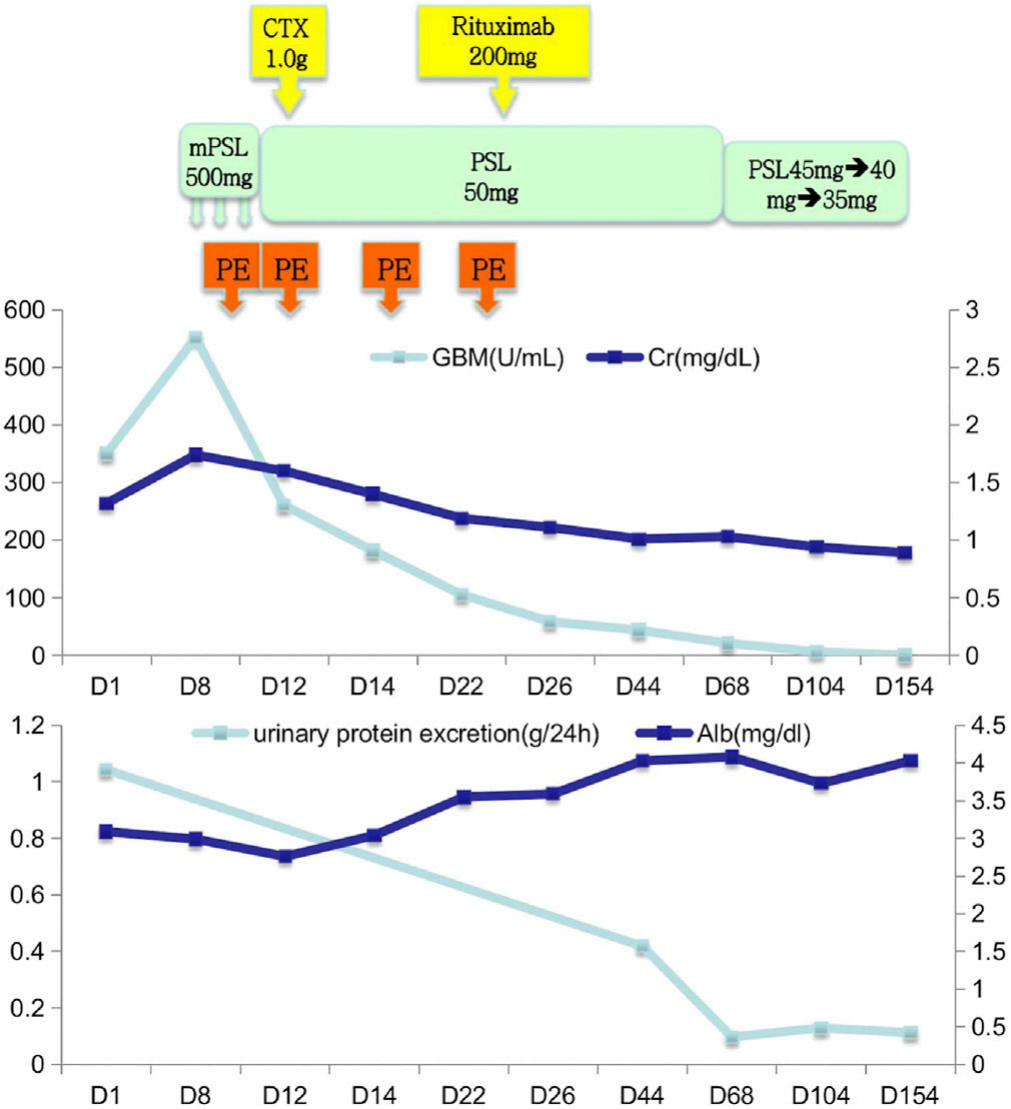
Clinical course. PSL: prednisolone, CTX: cyclophosphamide, PE: plasma exchange.

The patient was diagnosed with acute kidney injury, anti-GBM disease, MN at stage II, IgA nephropathy (Oxford classification in IgA nephropathy, M0E0S1T1-C0), and hypertension. Although classical linear deposition of IgG along the GBM was not observed, intensive immunotherapy was also administrated to the patient considering the high titer of the anti-GBM antibody and the significant danger of anti-GBM disease. Meanwhile, the MN and IgA nephropathy were also treated by intravenous infusion of methylprednisolone (mPSL) (500 mg) for 3 days, sequential combination therapy with prednisolone (50 mg) by daily oral administration in the morning, plasma exchange (four times), and intravenous infusion of cyclophosphamide (CTX) (1.0 g), and to alleviate the disease, we added rituximab (200 mg). On discharge, the anti-GBM antibody was detected at the titer of 58.50 U/mL (by ELISA), and the serum creatinine level was 1.1 mg/dl. Regular clinical follow-up was conducted after discharge, and the orally administrated dose of prednisolone was gradually reduced (Figure 4). Throughout the 5 months of follow-up, the titer of the anti-GBM antibody was found to decrease to a normal range, the serum creatinine level stabilized, and the quantitative urinary protein excretion and urine red blood cells gradually decreased to a normal level. The patient was generally in good condition.

## Discussion

Many cases have reported the coexistence of anti-GBM disease and MN. The GBM injury during the course of MN may induce the release of normal or altered GBM matter, thus producing the anti-GBM antibody (Klassen et al., 1974; Pettersson et al., 1984; Thitiarchakul et al., 1995). Zhang et al. established the MN model in the DBA/1 mice through immunization with a3 (IV) NC1 (Zhang et al., 2012). The mice developed the circulating antibody against the a3 (IV) NC1; the antibody is bound to the kidney while the mice show characteristics of MN. The a3 (IV) NC1 in the GBM is generally derived from podocyte (Abrahamson et al., 2009). When the anti-a3NC1 autoantibody is bound to a3 (IV), it may produce subepithelial immune complexes (Jia et al., 2014). At present, a dual mechanism has been proposed to elucidate the pathological physiology for MN occurring after an anti-GBM disease or concurrent MN and anti-GBM disease. In the first stage, the antibody is combined with antigenic structures anchored on glomerular capillary walls to produce linear deposition of IgG, thus promoting upregulation of basement membrane antigens synthesized and secreted by podocytes. In the second stage, multi-specific antibodies react with these basement membrane components to form immune complexes in the subepithelial areas *in situ* (Nasr et al., 2003).

Compared with the anti-GBM disease alone, patients with the coexisting anti-GBM disease and MN showed a narrower autoantigen spectrum for the circulating anti-GBM antibody. Only the α3 chain of type IV collagen was identified in the GBM from the serum of most patients with coexisting anti-GBM disease and MN, while multiple α chains of type IV collagen were detected in the serum of most patients with anti-GBM disease alone. Probably due to the decreased overall antibody reactivity in patients with the coexisting anti-GBM disease and MN (Jia et al., 2014), these patients demonstrated high retention of renal function (Hirayama et al., 2008). Jia et al. compared eight cases with concurrent anti-GBM disease and MN and 30 cases with anti-GBM disease alone and found that the former group was reported to have lower incidence rates of oliguresis, anuria, and gross hematuria. Compared with patients with anti-GBM disease alone, those with concurrent anti-GBM disease and MN excreted much urinary protein. At diagnosis, the serum creatinine level of patients with the anti-GBM disease alone was higher than that of patients with both diseases (Jia et al., 2014). The IgG1 and IgG3 subclasses of the anti-GBM antibody were found to influence the progression of renal injury in patients with the anti-GBM disease (Zhao et al., 2009). Troxell et al. demonstrated the relationship of the pathogenetic sequences of anti-GBM disease and MN with prognosis in terms of the kidney. Five patients with MN apparently prior to anti-GBM glomerulonephritis showed poor prognosis of kidney and progressed to end-stage renal disease. Of five patients, four with anti-GBM glomerulonephritis obviously prior to MN retained renal function (Troxell et al., 2006).

Superimposition of anti-GBM disease on latent IgA nephropathy can possibly explain the coexistence of anti-GBM disease and IgA nephropathy. Previous research also hypothesized that IgA-related immune complexes may promote immunologic and inflammatory events, causing conformational changes and exposure of GBM antigens and thus the development of the anti-GBM antibody (Trpkov et al., 1998). The abnormity of IgA molecules may be another factor in the pathogenesis of IgA nephropathy with the anti-GBM disease. Deposition of abnormal galactose deficient IgA1 antibody causes the formation of new GBM antigens, thus triggering autoimmune responses (Cui et al., 2006). Seasonal viral infections and repeated intestinal mucosal irritation are separately presumed to be parts of the pathological physiology of anti-GBM disease and IgA nephropathy and may be common links for the coexistence of the two diseases (Alchi et al., 2015). However, it is challenging to determine whether the anti-GBM disease in the patient is secondary to IgA nephropathy because there is still no established marker with which to distinguish primary from secondary anti-GBM disease.

Suh et al. found that compared with patients with the anti-GBM disease accompanied by immune complex deposition (40%), patients with the concurrent anti-GBM disease and IgA nephropathy reported fewer oliguria symptoms (10%). The percentage of glomeruli with crescents in patients with the concurrent anti-GBM disease and IgA nephropathy (59%) was lower than that in patients with the anti-GBM disease accompanied by immune complex deposition (93.8%) (Suh et al., 2019). Most patients with the combined anti-GBM disease and IgA nephropathy did not show lung involvement. According to an existing retrospective analysis of a limited number of cases, patients with the concurrent anti-GBM disease and IgA nephropathy had a better prognosis than those with anti-GBM disease alone or with anti-GBM disease accompanied by immune complex deposition.

IgA nephropathy complicated with MN has also been reported in previous research, which shows clinical and pathological features of both diseases. Patients with concurrent IgA nephropathy and MN exhibited similar clinical features to MN patients, while their severity was lower than that in patients with IgA nephropathy (Chen et al., 2018). Previous research suggested that patients with concurrent IgA nephropathy and IMN showed a lower proportion of segmental sclerosis (Chen et al., 2017) and better prognosis compared with those with MN alone.

Although it remains unclear which glomerular disease occurred first in the patient reported here, the preserved renal function indicates that the anti-GBM disease might occur before MN and IgA nephropathy might be latent for a longer time. There is also a probability that the patient shows a favorable prognosis due to being complicated with IgA nephropathy. In view of this, IF staining was performed for IgG subclasses, which mainly revealed IgG4 deposition and was positive for PLA2R, indicating PLA2R-associated MN. The serum anti-PLA2R titer level can reflect disease activity, and the PLA2R-associated MN with negative serum anti-PLA2R may be in the initial stage of the disease, with less damage to the glomerular filtration membrane and less urine protein. It also explains why the pathological type of the patient was MN but the level of proteinuria was low. Serum anti-PLA2R negativity may also be because the positive rate of serum anti-PLA2R was about 70.0%–80.0%. The staining was negative for IgG3 deposition, which might indicate a favorable prognosis with respect to the kidney. The three diseases may also have potential causal relationships, which warrant further in-depth research and analysis of more cases. In addition, the probability of concurrence of the three diseases also needs to be considered.

The use of plasma exchange and high-dose cytotoxic drugs to remove circulating antibodies and inhibit antibody production is a therapeutic strategy for anti-GBM diseases (McAdoo and Pusey, 2017). Cytotoxic therapy is generally limited to the short-term treatment for several months of anti-GBM disease due to the low recurrent risk of the disease. Immunosuppressive therapy of IgA nephropathy remains controversial and is limited to high-risk groups. More intensive or longer immunosuppressive therapies are probably necessary for patients with the concurrent anti-GBM disease and IgA nephropathy (McAdoo and Pusey, 2017). Rituximab has recently been used in some patients with anti-GBM diseases and significantly reduced the titer of the anti-GBM antibody, showing favorable clinical effects after treatment (Jain et al., 2018; van Daalen et al., 2018). Therefore, intensive immunosuppressive therapy was selected for the patient (considering the three diseases) and obtained satisfactory results.

## Conclusion

The research reports a patient with a concurrent anti-GBM disease, IMN, and IgA nephropathy. Although the pathological examination of the kidney did not suggest a classical presentation of the anti-GBM disease, plasma exchange and intensive immunotherapy were also used. The renal function and proteinuria improved with the treatment. The patient showed a favorable prognosis probably because it was complicated with IMN and IgA nephropathy. However, the potential links between the three diseases require further research.

## Data Availability

The original contributions presented in the study are included in the article/Supplementary Material. Further inquiries can be directed to the corresponding author.
